# *Streptococcus bovis* infectious endocarditis and occult gastrointestinal neoplasia: experience with 25 consecutive patients treated surgically

**DOI:** 10.1186/s13099-015-0074-0

**Published:** 2015-10-15

**Authors:** Anthony Alozie, Kerstin Köller, Lumi Pose, Maximilian Raftis, Gustav Steinhoff, Bernd Westphal, Georg Lamprecht, Andreas Podbielski

**Affiliations:** Department of Cardiac Surgery, University Heart Center Rostock, Schillingallee 35, 18057 Rostock, Germany; Institute of Medical Microbiology, Virology and Hygiene, University Hospital Rostock, Schillingallee 70, 18055 Rostock, Germany; Zentrum für Innere Medizin, Klinik II -Abteilung für Gastroenterology, Ernst-Heydemann-Str. 6, 18057 Rostock, Germany

**Keywords:** Infectious endocarditis, *Streptococcus bovis*, Colonic neoplasia

## Abstract

To assess the prevalence of gastrointestinal neoplasia in patients with *Streptococcus bovis* infectious endocarditis we performed a retrospective cohort analysis of all episodes of *S. bovis* infectious endocarditis treated at our institution between January 2000 through December 2014. Twenty-five patients were identified for this purpose. 12/25 patients received colonoscopy and 1/25 of the patients was assessed with CT colonography. Of the 13 who underwent colonic assessment, 11 were diagnosed with colonic neoplasms at different stages of development. In the absence of any strong contraindication, gastroenteroscopic evaluation in all patients diagnosed with *S. bovis* infectious endocarditis should be pursued.

## Background

*Streptococcus bovis* (*S. bovis*), is a commensal inhabitant of the human digestive tract. It may be isolated in up to 35 % of fecal samples from human rectal swab [1]. Since the first remarkable case of the association between an enterococcal infection and colorectal carcinoma presented by McCoy et al. in 1951 and the case control study by Klein et al. over two decades later, the association between *S. bovis* bacteremia and colorectal neoplasia has been thoroughly well established in the literature [2–4]. The involvement of *S. bovis* in infectious endocarditis (IE) was put at approximately 6 % [5, 6], which means that IE is another important variable associated with the two phenomena. A systematic review of 31 studies published by Boleij et al. in 2011 found that 65 % of patients infected by *S. bovis,* in addition to high levels of IE, were also diagnosed with concomitant colorectal neoplasias [7]. The picture became more complex, because genomic analysis revealed that “*S. bovis*” isolates in fact belonged to at least seven different species or subspecies, i.e. *S. gallolyticus* ssp. *gallolyticus*, *S. gallolyticus* ssp. *pasteurianus*, *S. gallolyticus* ssp. *macedonius*, *S. infantarius* ssp. *infantarius*, *S. infantarius* ssp. *coli* [*S. lutetiensis*], *S. alactolyticus*, and *S. equines.S. gallolyticus* ssp. is more often encountered in human specimens than the two other species/subspecies. Especially *S. gallolyticus* ssp. *gallolyticus* bacteremias were demonstrated to be associated with colorectal cancer [8, 9]. For reasons of simplicity these strains are furtheron addressed as *S. bovis*-group isolates.

Reported colorectal neoplasia encompassed adenomas with neoplastic potential, which included tubular, tubulo-villous and villous adenomas. Given this relationship between *S. bovis*-group bacteremia and colorectal neoplasia, patients with IE treated conservatively or surgically should be promptly evaluated for presence of gastrointestinal neoplasia, since colorectal neoplasia seem to be the preferred colonization site for *S. bovis*-group strains [7].

## Methods

Etiologic diagnosis of *S. bovis*-group IE in 25 patients treated surgically between January 2000 and December 2014 in our heart center was obtained by blood culture and direct valve culture results. Routine 16 sRNA PCR of all intra operatively excised heart valves from IE patients was introduced from year 2007. Blood culture isolates from external hospitals and those obtained prior to 2007 were not further characterized by molecular genetic methods (8 isolates). However, full-length 16 sRNA PCR gene sequences of blood and heart valve cultures obtained during the later years clearly assigned the strains to *S. gallolyticus* subsp *gallolyticus* (16) and *Streptococcus lutetiensis* (1). Clinical information was obtained from the patient clinical record. This included presenting symptoms, examination findings, and investigation results from echocardiography, abdominal sonography, computed tomography for general screening purposes and gastroscopy/colonoscopy. Clinical records were reviewed for data regarding demographics, medical co-morbidities, clinical presentation, investigations, surgical interventions, clinical outcome and follow up management. A questionnaire was submitted to each patient’s general practitioner to assess current health status. A telephone interview was made if the questionnaire was not returned.

Diagnosis of IE was established by a team of cardiologists, heart surgeons, microbiologists and pathologists applying the modified Duke criteria [10].

### Microbiology

Processing of blood cultures followed the established standards of the German Society for Hygiene and Microbiology (DGHM). The microbiology laboratory of Rostock University Medicine is accredited according to DIN EN ISO 15183 for these tests as well as for the PCR examinations. DNA extraction was performed with the Qiagen DNA Mini Kit (QIAGEN, Hilden, Germany) according to the manufacturer’s protocol. Nucleic acid concentration was measured using a biophotometer (Eppendorf, Hamburg, Germany). For 16S rDNA PCR primers, 16S8_27 and 16S_907 and polymerase moltaq (molzym) were utilized [11, 12]. The following reaction conditions were chosen: (1) 15 min at 94 °C; (2) 30× [1 min at 94 °C, 1 min at 50 °C, 1 min at 72 °C]; (3) 5 min at 72 °C [11, 12]. PCR products were determined by gel electrophoresis, purified with the NAT CLEAN-UP/NUCLEOSPIN^®^ EXTRACT II (Machery-Nagel, Düren, Germany) and subsequently sent to Microsynth Sequencing Device (Göttingen, Germany) for the actual sequencing reaction. Sequence analysis was performed using NCBI nucleotide blast search (http://www.ncbi.nlm.nih.gov), resulting in identification of various species of the *S. bovis*-group (Table 1).

**Table 1:** Patients baseline characteristics, gastrointestinal findings and underlying conditions

Pat. nr	Age/sex	Year	Bio-type	IE	Bowel evaluation	Findings	Underlying conditions
1	64/m	2003	*S. bovis*	Definite	Gastroscopy, colonoscopy	Two 1–1.5 cm tubular sessile adenoma, intermediate dysplasia	CLL, microcytic anemia, BPH, carcinoma of prostate 3 years later
2	49/m	2004	*S. bovis*	Definite	Declined	Colosigmoid adenocarcinoma 5 years later	Alcohol toxic liver cirrhosis, esophagus varices III°
3	69/m	2004	*S. bovis*	Definite	Gastroscopy, colonoscopy	Gastritis	Gastritis, colostoma (post sigma ishemia)
4	49/m	2005	*S. gallolyticus* subsp*. gallolyticus*	Definite	Colonoscopy	Two 1 cm tubular colon adenoma low grade dysplasia	None
5	64/m	2007	*S. gallolyticus* subsp*. gallolyticus*	Definite	Colonoscopy	Five 1–2 cm polyps, with high grade suspicion for coecum carcinoma	None
6	72/m	2007	*S. gallolyticus* subsp*. gallolyticus*	Definite	No colonoscopy	–	None
7	53/m	2008	*S. gallolyticus* subsp*. gallolyticus*	Definite	No colonoscopy	–	None
8	70/m	2008	*S. gallolyticus* subsp*. gallolyticus*	Definite	No colonoscopy	–	Hepatitis C, PEG interferone, dialysis
9	73/m	2009	*S. gallolyticus* subsp*. gallolyticus*	Definite	Colonoscopy	2 × 2 cm tubular Polyps, high grade suspicion for carcinoma 2014	Liver cirrhosis, psoriasis
10	54/m	2009	*S. gallolyticus* subsp*. gallolyticus*	Definite	Gastroscopy, colonoscopy	Pangastritis, 2× broad based colonic adenoma	Pangastritis
11	70/m	2009	*S. gallolyticus* subsp*. gallolyticus*	Definite	Lost to follow up	–	Spinal abscess surgery
12	57/m	2009	*S. gallolyticus* subsp*. gallolyticus*	Definite	Colonoscopy	Diverticulosis of small/large intestine	
13	74/m	2010	*S. gallolyticus* subsp*. gallolyticus*	Definite	Colonoscopy/gastroscopy	Broad based stomach Polyp	Spondylodiscitis
14	66/m	2010	*S. gallolyticus* subsp*. gallolyticus*	Definite	No colonoscopy	–	None
15	77/m	2011	*S. bovis*	Definite	CT colonography	Normal	Steatosis hepatis, BPH
16	48/m	2012	*S. gallolyticus* subsp*. gallolyticus*	Definite	Colonoscopy	4 Colonic polyps, 7–15 mm; stomach polyp, mucus-producing tumor left colonic flexure	Liver cirrhosis child B, esophagus varices II°
17	60/m	2012	*S. gallolyticus* subsp*. gallolyticus*	Definite	No colonoscopy	–	None
18	63/f	2012	*S. gallolyticus* subsp*. gallolyticus*	Definite	No colonoscopy	–	None
19	68/m	2013	*S. gallolyticus* subsp*. gallolyticus*	Definite	Colonoscopy	Colonic polyp	None
20	57/m	2013	*S. gallolyticus* subsp*. gallolyticus*	Definite	No colonoscopy	–	Hepatitis B, chronic alcohol abuse
21	84/m	2000	*S. bovis*	Definite	Colonoscopy	Multiple colonic polyps up to 1 cm each	Cholecystolithiasis, splenomegaly
22	65/m	2000	*S. bovis*	Definite	No colonoscopy	–	Ethyl toxic liver insufficiency, esophagus varices III°
23	78/m	2014	*S. lutetiensis*	Definite	Colonoscopy	Colonic and rectal polyps resected	None
24	40/m	2000	*S. bovis*	Definite	No colonoscopy	–	Hepatitis C and HIV
25	74/f	2007	*S. bovis*	Definite	No colonoscopy	–	Dialysis due to cystic kidney disease

*CLL* chronic lymphocytic leucemia, *BPH* benign prostate hyperplasia, *PEG-interferone* pegylated interferone

## Results

Twenty-five *S. bovis*-group IE episodes with available patient clinical data were identified. These were in 23 males and 2 females. Mean age was 63.9 (range 40–84 years).

A definite IE according to the duke criteria was established preoperatively in all patients and confirmed by post surgical histological and microbiological processing of every single excised heart valve. Sixteen of the IE episodes were caused by *S. gallolyticus* subsp. *gallolyticus* and the remaining nine cases by 1 *S. lutetiensis* and 8 isolates of the *S. bovis*-group with no further differentiation.

Of the 25 patients examined, 13 received gastroenteroscopic assessment for gastrointestinal neoplasia post surgery by means of gastroscopy, colonoscopy and ct colonography. Of these 13 patients, 11 had gastroentrologic neoplasia (84.6 %). Detailed evaluation of gastroenteroscopic findings in 11 of the 13 patients assessed are depicted in Table 1. One patient with ethyl toxic liver cirrhosis and esophagus varices grade III° at time of surgery who refused colonoscopy was diagnosed with colo-sigmoid carcinoma 6 years later.

## Discussion

The clinical relationship between *S. bovis*-group bacteremia and underlying gastrointestinal malignancies or premalignant adenomatous polyps has been well known for many years [2, 3]. Although this association has huge clinical implications for both patients and physicians being entrusted with further management of these patients both during major illnesses and thereafter, we found unsatisfactory awareness of this among family doctors. This is reflected in the unacceptably low number of patients we observed in this study obtaining gastroenteroscopic evaluation after hospital discharge. Those patients who did obtain gastroenteroscopic evaluation, only received this during their hospital stay. Of those who were discharged without gastroenteroscopic evaluation, lack of compliance was often noted, but the remaining patients were probably not followed up as required. One patient in our series was diagnosed with colonic adenocarcinoma 6 years later, after he had refused gastrointestinal evaluation post surgery. We speculate that this occurrence could probably have been prevented if a colonoscopy had been obtained in the first place.

The rate of patients undergoing colonic evaluation in our study (52 %) did not significantly differ from those reported in previous literature [13, 14] (70 and 50 %) although it was far lower than those reported by Ballet et al. (81 %) and Tripod et al. (96 %) [15, 16]. However, the prevalence of colonic malignancies or premalignancies we found in those patients subjected to gastroenteroscopic evaluation 13/25 is in between those reported in other studies, with a similar number of patients subjected to colonic evaluation (33 %, Coffey and 86 % Vaska) [13, 14]. *S. gallolyticus* subsp. *gallolyticus* has a median colorectal neoplasia prevalence among *S. bovis* bacteraemia patients of 60–67 % but only 25 % prevalence in the general population that underwent colonoscopy, showing the clear association of *S. gallolyticus* subsp. *gallolyticus* with colorectal neoplasia [9]. Current medical recommendations therefore advice to perform colonoscopy for any patient diagnosed with *S. gallolyticus* subsp. *gallolyticus* bacteraemia due to the 2/3 association of *S. gallolyticus* subsp. *gallolyticus* with colorectal neoplasia and 21 % yielding de facto colorectal carcinomas [9].

Of all 13 patients in our study that obtained colonic evaluation, 11 had colonic malignancies or premalignancies (84.6 %). Since our study is neither randomized nor prospective in nature, we cannot exclude the possibility that these results are obtained by chance. However, our results are in line with other studies with high prevalence of malignancies or premalignancies of the colon with the same setting [13]. We admit that there is a wide range between reported prevalence of gastrointestinal neoplasia during *S. bovis* bacteremia, ranging from 6 % in the retrospective analysis presented by Pigrau et al. to 86 % as reported in a retrospective study by Vaska et al. [17]. This may be partly due to the retrospective nature of these studies, as a limited amount of patients underwent bowel evaluation after episodes of bacteremia.

## Conclusion

Adenomatous polyps are tumors of benign neoplastic epithelium with variable potential for malignancy. The adenoma-carcinoma sequence is well known and it is accepted that more than 95 % of colorectal cancers arise from adenomas [18]. Though, to date there has been no satisfactory explanation regarding the pathophysiological mechanism behind this association. We admit that we were not able to provide precise differentiation between all species and subspecies, a factor necessary for correct risk assessment of every diagnosis. In consideration of this fact, we strongly advocate subtyping of every single case of *S. bovis* bacteraemia and gastroenteroscopic assessment in all patients diagnosed and treated for *S. bovis*-group IE during or soon after their index admission, as 84.4 % of our patients who obtained this assessment had colorectal neoplasia.
